# The feasibility of integrating a home telehealth model for older persons living with hemodialysis

**DOI:** 10.1186/s12877-024-04981-8

**Published:** 2024-04-27

**Authors:** Wanicha Pungchompoo, Saowaros Parinyachitta, Sirirat Pungchompoo, Warawan Udomkhwamsuk, Panadda Suwan

**Affiliations:** 1 Department of Medical Nursing, Faculty of Nursing, Chiang Mai University, Chiang Mai, Thailand; 2 Department of Nephrology, Faculty of Medicine, Chiang Mai University, Chiang Mai, Thailand; 3 Department of Industrial and Manufacturing Engineering, Faculty of Engineering, Prince of Songkla University, Songkhla, Thailand; 4 Department of Surgical Nursing, Faculty of Nursing, Chiang Mai University, Chiang Mai, Thailand; 5 Department of Medicine, Faculty of Medicine, Chiang Mai University, Chiang Mai, Thailand

**Keywords:** Older persons, Hemodialysis, Home telehealth model

## Abstract

**Background:**

In Thailand, there is a rapidly increasing population of older persons living with hemodialysis (OPLWH) for whom quality of life and clinical outcomes are their main focus. This study aims to assess the feasibility of an integrated home telehealth model on quality of life and laboratory parameters of OPLWH.

**Methods:**

In this study, the second phase of a mixed methods exploratory sequential design was conducted using a repeated measures experimental design. Participants met the inclusion criteria, which included being an OPLWH at a single hemodialysis center of one hospital in Chiang Mai province, Thailand, during the experimental period between 1 April and 30 September 2018, and willing to participate in the study. The 54 participants were purposively selected and randomly assigned to receive either an intervention (*n* = 24) consisting of health education and health monitoring using a telehealth device (an iPad) and a web application, or usual care (*n* = 30). The instruments included a demographic data form, which was analyzed using the chi-square test. The health-related quality of life questionnaire (the 9**-**item Thai Health Status Assessment questionnaire) and blood chemistry (BUN, Cr, Hb, Hct, Alb, K, Kt/V, and nPCR) were compared and measured at baseline, and at 3 and 6 months after enrolment using independent t-test and one-way repeated measures ANOVA.

**Results:**

The comparison of quality of life between the two groups at the two points of repeated measurement (after 3 months) showed a statistically significant difference in mental health scores at *P* < *0.05*. Six months after the intervention, mean scores for health outcomes and patients’ quality of life improved; however, this change did not reach statistical significance.

**Conclusions:**

An integrated home telehealth model implemented by a hemodialysis nurse is a feasible holistic care approach for OPLWH. However, the absence of statistical significance may partly be associated with the clinical characteristics of frailty and risk factors such as increased age, hypertension, diabetes, heart disease, longer dialysis time, and inadequacy of Kt/V. Large-scale multi-centre trials are warranted to fully examine the acceptability of the model. The duration and long-term effects of the telehealth model are also recommended for further investigation.

**Patient or public contribution:**

The development of a home telehealth model was a collaborative process between patients, caregivers, healthcare professionals from the hemodialysis unit, and the research team.

## What is already known?

- In the wake of the COVID-19 pandemic, we are living in a new normal wherein many telehealth systems have been developed—for example, the use of telemedicine to prevent COVID-19.
- However, little is known about whether an integrated home telehealth model might be able to promote quality of life and prevent complications for older persons undergoing hemodialysis.

## What does this paper contribute to the wider global clinical community?

- This paper outlines the development of a home telehealth model specifically focused on holistic care for OPLWH.
- The study demonstrates increases in health-related quality of life across both physical and mental scores as a result of receiving the telehealth model. Six months after the experimental intervention, there was no statistical difference between the two groups, but the evidence revealed positive changes in mean scores for quality of life and some laboratory results compared with participants who received usual care.
- An integrated holistic home telehealth model delivered by renal nurses and with interprofessional management is recommended as a feasible new clinical care model for the management of OPLWH at home. This is the first model of its kind to be implemented in Thailand.

## Introduction

The structure of the world’s population is changing, with an increase in the proportion of older persons. The proportion of the global population aged 65 years or above is projected to rise from 10% in 2022 to 16% in 2050. By 2050, the number of persons aged 65 years or over, worldwide, is projected to be more than twice the number of children under age 5, and about the same as the number of children under age 12 [1]. The number of Thai older people has increased seven-fold in recent years, and by 2035, is forecast to reach 20 million [2].

More than 3 million Thais aged 60 and over have been living with chronic illness during the past five years, a statistic that illustrates the increase in chronic illness among the elderly population [3]. Chronic kidney disease (CKD), a progressive chronic disease common in older persons, is estimated to affect approximately 44% of individuals aged 65 and older [4] and more than 10% of the population worldwide [5, 6]. End-stage renal disease (ESRD) refers to patients with an estimated glomerular filtration rate lower than 15 ml per minute per 1.73 m^2^ body surface area [7, 8]. ESRD treatment involves renal replacement therapy (RRT), which includes peritoneal dialysis (PD), hemodialysis (HD), and kidney transplantation (KT) [9]. New cases of Thais living with end-stage renal disease (ESRD) and receiving hemodialysis reached 39,398 during the period from 2017 to 2019 [10]. Patients receiving hemodialysis include those aged 45 to 64 years (41%), 65 to 74 years (24%), and 75 years and over (20%) [10]. Older persons undergoing dialysis with ESRD represent a major health and social care burden in the context of aging populations [4].

Considering this situation, nurse-led telehealth could contribute to managing some of these problems by using information and communication technologies [11]. Telehealth is a broad term encompassing the use of electronic communication to provide clinical care by replacing face-to-face visits; however, the focus here is on interactive videoconferencing, remote monitoring, mobile phones, web applications, and the internet of things [12]. Telehealth, integrating holistic and interprofessional management, is a new model of care for chronic disease management. The use of telehealth to facilitate the assessment of symptoms reported by patients themselves may also enhance effective symptom management for persons with end-stage renal disease and could provide a means to overcome identified barriers to home care, thereby improving patients’ care experiences [13, 14].

Home telehealth can be used to deliver a range of interventions, from providing information to supporting therapeutic procedures. More timely visits may also result in earlier interventions, preventing complications and reducing unnecessary use of health services while assisting patients to manage their symptoms at home [15]. Patients who are ill or who find it difficult to travel to attend appointments with physicians can benefit from reduced travel costs; hemodialysis treatment often requires time off work and time away from family, in addition to the financial burden of transportation [12]. Remote monitoring of patients through information and communication technologies, termed “telecare” or “telehealth”, is increasingly being evidenced as a means of addressing the interest in and demands on health services, alongside more patient-focused care [16].

**Fig. 1:**
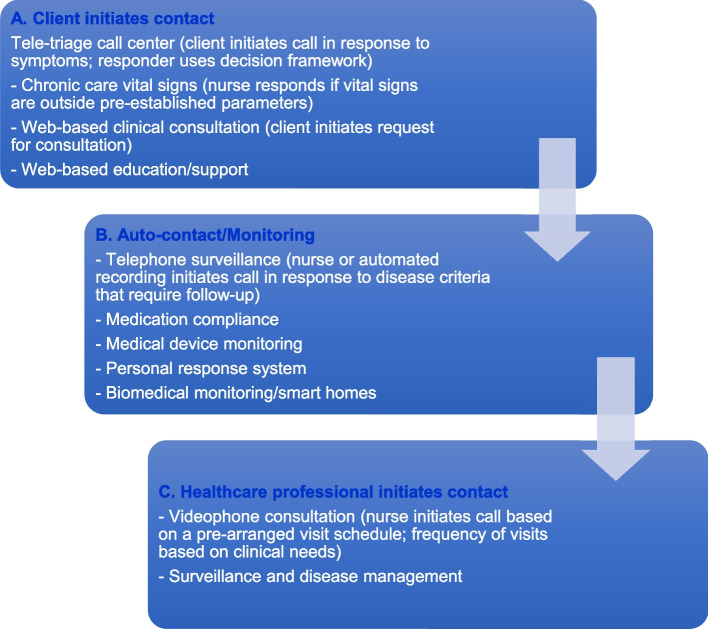
Continuum of home telehealth technologies (adapted from Hebert, Korabek, and Scott, 2006, p. 788) [15]

The conceptual framework of this study applies the continuum of home telehealth technologies, developed by Hebert, Korabek, and Scott, to examine the feasibility of using a home telehealth program for older persons living with hemodialysis (OPLWH) in Thailand [15]. These technologies can be categorized following the 3 steps of the home telehealth technologies’ continuum [17], from 1) patients initiating contact, through 2) automatic monitoring via technology, to 3) healthcare professionals initiating contact (see Fig. 1). Categorizing telehealth services may also be based on desirable patient outcomes and the need for services [15]. The combination of effective telehealth and a continuum of healthcare will increase understanding of health, patient outcomes, and the cost of care. Previous studies have suggested that interprofessional care management or home monitoring could improve intermediate outcomes of CKD patients [13].

The effectiveness of telehealth lies in its potential for improving symptom management and quality of life for patients with long-term chronic conditions, as well as potentially reducing healthcare costs; these benefits have been demonstrated in a number of recent studies [9, 12, 14, 18]. Telehealth has also been successfully implemented to encourage meaningful engagement with the care system, especially for primary outcomes among both rural and urban patients with chronic kidney disease (estimated glomerular filtration rate (eGFR) < 60 ml/min/1.73m^2^). Telehealth and the remote monitoring of older patients living with chronic conditions may also provide a means to address geographical barriers to continuum care, thereby improving access to home dialysis, patient quality of life, and outcomes [12]. Furthermore, the impact of telehealth interventions on clinical outcomes can be reported through lower hospitalization rates and fewer clinical visits. In addition, it has been found that patients who receive telehealth were less likely to miss HD treatment sessions compared with patients who received standard care [19]. A one-year randomization control trial study, with baseline characteristics of participants including a mean age of 75.1 ± 8.1 (SD) years, found that the telehealth implementation of the Patient Aligned Care Team (PACT) was independently related to reductions in emergency department visits and hospitalization, while improving the ability to control hypertension [14]. In addition, a longitudinal cohort study confirmed that telehealth videoconferencing for kidney transplant and CKD patients with a mean age of 63.9 years (SD = 12.3 years) provided a feasible intervention for 1 year and sustainable outcomes [20]. Another qualitative study explored the perceptions of telehealth of older adults with advanced CKD, their partners, and kidney clinicians, demonstrating that telehealth may improve convenience and care partner engagement while still presenting concerns about clinical effectiveness, limitations of virtual physical examination, reduction of the patient-clinician relationship, and patient trust [21].

During the last decade, the number of OPLWH in Thailand has been increasing steadily. While improving patient outcomes and achieving higher quality care at a lower cost are two goals of adopting telehealth technology, it is necessary to better understand the underlying rationale for how and why home telehealth is effective for improving health outcomes and reducing care costs.

### The study

The overarching mixed-methods research project of which this study is a part was conducted to explore and examine the feasibility of using home telehealth for older patients living with end-stage renal disease. We conducted this study as phase 2 of a project entitled Integrating home telehealth into the holistic care: Pilot study in older persons with ESRD to examine whether integrated home telehealth with nurse oversight could be effectively implemented and whether it could improve the quality of life and clinical outcomes in OPLWH when compared with usual care.

## Methods

A mixed-methods exploratory sequential design was conducted for the project entitled Integrating home telehealth into the holistic care:

Pilot study in older persons with ESRD. Mixed-method research is the combining and integrating of two research types, namely qualitative and quantitative research [22, 23]. A description of the study carried out as phase 1 of the project has been published previously [24]. In this manuscript, we report on phase 2 of the study.

**Fig. 2:**
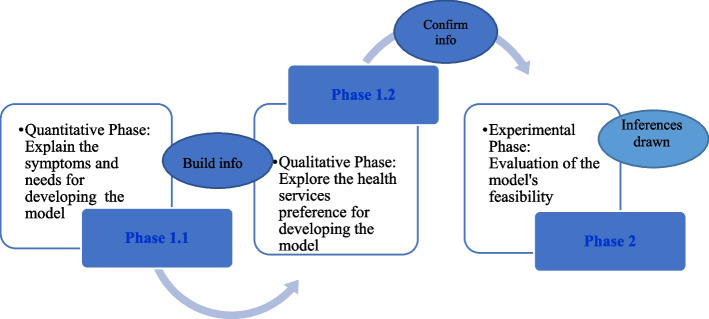
The scope of the study design

Building on the exploratory results from phase 1, this second phase focuses on gathering quantitative data in order to test or generalize the initial findings. Interaction between the qualitative and quantitative strands occurs as the researcher develops a telehealth model as an intermediate step between the two phases, building on the qualitative results and using them in the subsequent qualitative data collection. The exploratory sequential design in two phases which has been used in this study is set out below, and the scope of the research is presented (see Fig. 2).

### Study design and setting

The first phase of the wider mixed-methods study aimed to describe and explore symptom experiences and needs related to the integration of home telehealth into holistic care with nurse oversight for OPLWH. A survey study design with a nonprobability sampling method was employed, and OPLWH were surveyed over a six-month period (between 1 January and 30 June 2017). Participants were recruited from two hemodialysis units at two hospitals in Chiang Mai province. Purposive sampling was used to identify and recruit the participants. The instruments included the VOICES (View of Informal Carers Evaluation of Service-ESRD/Thai patients’ version) questionnaire. Descriptive statistics were used to analyse the data.

Most participants had comorbid conditions, such as hypertension or diabetes mellitus and three main physical symptoms were found (shortness of breath, and pain, and edema) while two psychological symptoms were also present (anxiety and moderate stress). Participants had experienced readmission to hospital at least twice per month. Furthermore, the majority were unable to access home care.

Next, the qualitative section of the study was conducted via in-depth interviews with 20 participants to explore the needs of participants using thematic analysis to analyze the data. The findings revealed the needs of participants related to telehealth including four dimensions: 1) symptom management at home; 2) activity and role management; 3) emotional management; and 4) spiritual support [24]. The results of the first phase were then utilized to inform and develop an effective home telehealth program for OPLWH.

### Participants

In the second phase of the project, a repeated measures experimental design was implemented. Potential participants included 141 OPLWH aged 60 or over who were recruited from a single hemodialysis center at Maharaj Nakorn Chiang Mai hospital in Chiang Mai province, Thailand, during the period between 1 January 2018 and 30 March 2018. Finally, 54 participants met the inclusion criteria and were willing and able to participate in the study, as well as being capable of providing informed consent.

#### Inclusion and exclusion criteria

Participants were eligible to participate in the study if they were aged 60 years or over, and if they: 1) were being managed for hemodialysis and 2) had been diagnosed with end-stage renal disease stage 5 by a nephrologist. Eligible participants gave their informed consent to participate. Those who were hospitalized with acute illnesses, who had psychological or cognitive disorders, or who had physical limitations which affected their self-care were excluded. Those who were unable to meet the inclusion criteria or were not willing to participate were also excluded.

#### The sample calculation and randomization procedure

**Fig. 3:**
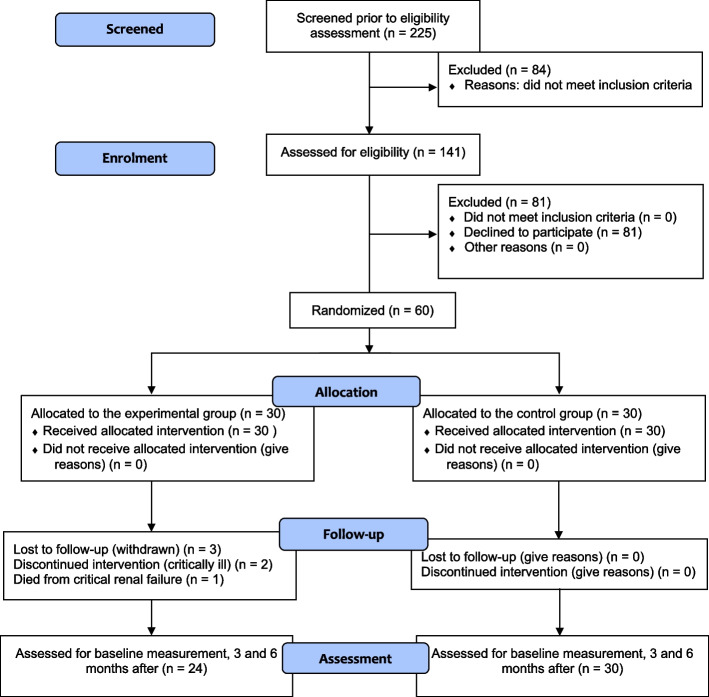
Recruitment flowchart

A simple randomization method using a 1:1 ratio was used to assign participants to either the experimental group or the control group, using the random allocation concealment method. Randomization was carried out over the telephone by the researcher who was blinded to patient identity. Although the participants could not be masked in relation to their assignment, the renal nurses caring for them were blind to the participants’ study assignment. Based on repeated measures analysis of variance, an estimated sample size of 60 (30 per group) was considered adequate to demonstrate the effects of the experimental intervention, according to previous studies [25, 26]. However, 6 participants in the experimental group were removed because they either withdrew (3), were admitted to the intensive care unit due to co-morbid conditions (2), or died from critical renal failure (1). Thus, 54 participants remained in the study: 24 in the experimental group, and 30 in the control group. G*Power software was used to determine the sample size, and the 8 study factors were analyzed using multivariate statistics. In general, 5–20 samples per factor are preferred. However, in this study, T-test and ANOVA statistics were used. In this determination, the degree of freedom of the factor levels was 2 (3 levels of a changing time period) with two groups (a control group and an experimental group), and the patrial eta square was 0.35 according to the ANOVA results. The sample size which was calculated varied from 24 to 27 samples when we changed the number of covariance from 1 to 12 covariances and used a power of test value of 0.85. Therefore, the results showed that the sample sizes were sufficient for analysis with the ANOVA method [27]. The attrition rate for the study was 14.58% [25] (Fig. 3).

### Intervention measures

#### Experimental group

##### Home telehealth model

**Fig. 4:**
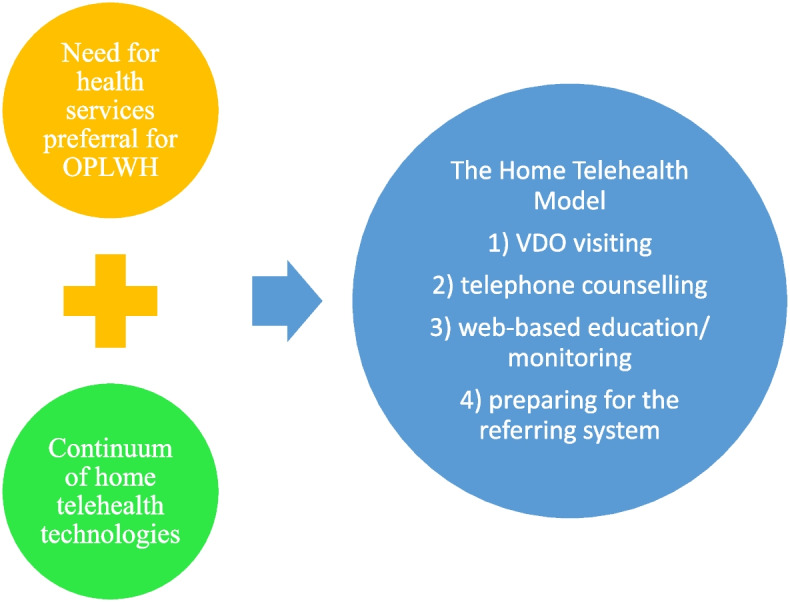
Framework of the holistic home telehealth model for OPLWH

The home telehealth model for OPLWH integrates two concepts. The first focuses on the requirement for health service provision for older persons related to their need for: the control of physical health symptoms, such as breathlessness, pain or edema; mental support and alleviating factors, such as anxiety and stress; spiritual support; clinical care, such as symptom management at home; psychological and spiritual support; regular home visits; an effective referral system; health education; and financial support [7, 24]. The second relates to the continuum of home telehealth technologies [15] (see Fig. 4). The framework for our holistic home telehealth model for OPLWH operates across four dimensions: 1) health education related to chronic kidney disease and treatment; 2) the referral system; 3) online home visits; and 4) telephone/live counselling.

The home telehealth model was designed and developed based on results from the first phase of the study [24]. The model was focused on video visiting, telephone counselling, web-based education and monitoring, and preparing for the referral system.

**Fig. 5:**
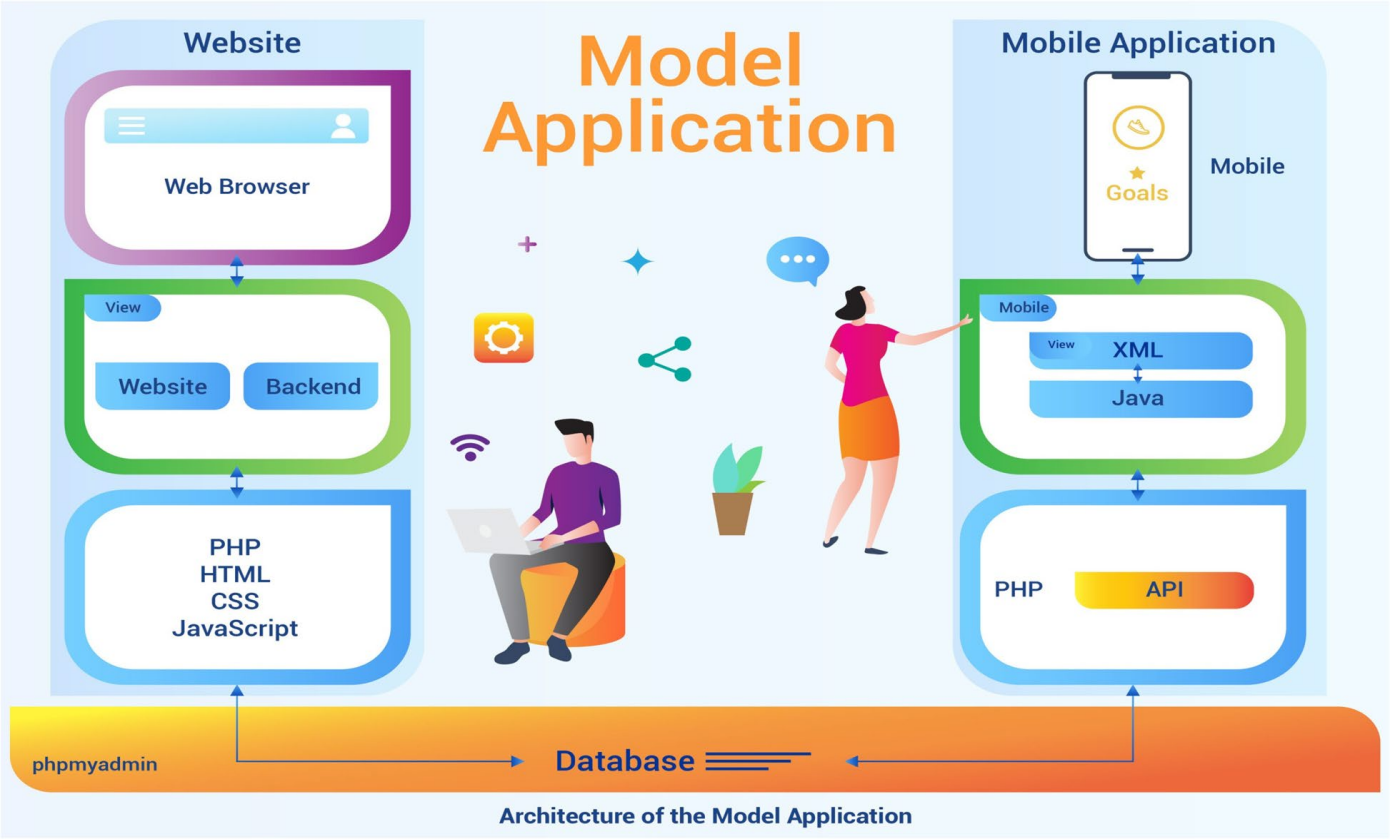
Data architecture of the model application

Five educational videos and a booklet were developed for participants, containing information and instructions about renal disease and treatment, food and water management, exercise and stress management, and medication management. A panel of experts made up of two renal nurse specialists, a nephrologist, and two experts in computer science evaluated the knowledge that was provided in the videos and the booklet for content validity and calibrated the web application system by testing the function of the model application’s data architecture (Fig. 5). This was combined with the website, which included a home page, a user panel, and an administration panel, and the mobile app, which included a user area and an expert area. The panel of experts made comments and suggestions, and the model was revised twice and returned to the panel of experts for evaluation until they were satisfied.

Finally, 3 research assistants, including an advanced practitioner nurse in hemodialysis and two hemodialysis nurses, were trained as “supporters” for assisting participants in the experimental group and also helping the researcher in implementing the intervention. The training session lasted for seven hours (a full day of training at the dialysis unit) and included a study information overview, information about the objectives and conceptual framework of the study, the instruments for recruiting potential participants, and the strategies applied to implement the model. Additionally, time was dedicated to sharing ideas, demonstrations, repeat demonstrations, and practice.

Participants and their caregivers in the experimental group received the home telehealth model in addition to standard care and were provided with a set of 1 manual and a tablet with internet connection for 6 months, containing the downloaded web application for the home telehealth model. They underwent a day of group teaching sessions, consisting of answering questions and addressing concerns as well as training in self-management skills. During each session the participants were educated about renal disease and treatment, food and water management, exercise and stress management, and medication management at home. Additionally, the participants and their caregivers were taught how to use the application, such as how to log in to the system, fill in their illness health history, record their body weight, make an online appointment, connect to Facebook, access Line to have a conversation with a hemodialysis nurse, and create a group chat with other patients. They also learned how to use video visiting, online monitoring, and telephone counselling.

The first week of group training sessions were followed by their six-month in-person participation in the home telehealth model, as well as routine online visits and telephone consulting, resulting in a total of one day per month for meeting and discussion with a hemodialysis nurse at the dialysis unit, and six months of monitoring and consultation online.

### Application of the home telehealth model for OPLWH: web application

The development of the home telehealth model resulted in an application that could be used on a website or on a mobile phone and is referred to here as the web application. The website and mobile versions are connected by an application programming interface. The website creates an application programming interface and receives orders from the application. The application will then send a request for interpretation and a summary.

At the same time, the home telehealth model website is a content management system that is divided into three parts including a home page, a user panel, and an administration panel. The patient’s data from the administration panel and the web page are recalled when the patient logs in to the website linked to the user panel. The mobile application is designed to be used on a mobile phone, tablet, or iPad by connecting data from the website with the application programming interface. The mobile application site consists of two sections (user and expert). The user section is for patients to log in to make appointments and view their health history. The expert section is connected to the application programming interface, Facebook Messenger, and is controlled by a nurse or physician who checks the online appointment requests made by patients. In this section, the nurse or physician can also provide online individual counselling or group discussions (Fig. 5).

#### Control group

The control group received standard care from the nursing staff at the renal unit. Standard care for persons receiving hemodialysis in government hospital in Thailand includes routine laboratory investigations, physical examinations, health education, consultations by a renal nurse and/or a general nurse, and monthly follow-ups. After finishing the study, the participants in the control group were invited to join the home telehealth model and received copies of the set of manuals on how to manage their hemodialysis at home.

### Measurement and data collection

#### Instruments and outcomes

##### The 9-item Thai Health Status Assessment Instrument

was used to evaluate the quality of life and health status of the dialysis patients [28] and is separated into two scale scores measuring physical and mental health. The instrument is composed of seven domains and two global health ratings [28, 29]. The seven domains encourage subjects to rate their experiences with health conditions during the past month. Response choices for the seven domains are interpreted according to the perceived severity of the problems on a 5-point scale, where 1 = “very severe”, 2 = “severe”, 3 = “moderate”, 4 = “mild”, and 5 = “not at all”.

The first global question is used to compare participants’ health at the present time with their health during the preceding year. The second question compares their health with the health of others of similar age, gender, social/economic status, employment type, and lifestyle. Response choices for the two global questions included 1 = “much worse”, 2 = “a little bit worse”, 3 = “the same as”, 4 = “a little bit better”, and finally 5 = “much better”. The calculated score values range between 20 and 80 (± 3SDs); if the physical or mental health score is above 20, the scores are interpreted as equal to the general healthy Thai population. Higher calculated scores reflect better health than the general population.

The 9-item Thai Health Status Assessment Instrument is a valid and reliable quality of life measure for Thai renal replacement therapy patients. Its convergent and divergent validity have been demonstrated using the SF-36 as a concurrent measure. Its concurrent validity has also been assessed using clinical variables. Its test–retest reliability is satisfactory as the inter-class correlation coefficients are 0.79 (physical health score) and 0.78 (mental health score) [25, 29].

After obtaining permission, we assessed the appropriateness and applicability of these instruments for our participant population. All the instruments were given to the participants in Thai language.

##### Demographic data

was collected at baseline. The participants were asked to provide information about their age, gender, marital status, educational level, occupation, and household income by completing the demographic data form. Also, the participants were asked about the duration of their hemodialysis treatment, while blood samples, and data related to their health status and health-related quality of life were collected at baseline (the firs week), 3 months (the last week), and 6 months (the last week).

##### Laboratory parameters

The laboratory parameters measured the effectiveness of the home telehealth model for older persons living with hemodialysis by comparing the significant differences between the experimental and control groups before the experiment, 3 months after, and 6 months after. The blood chemistry analyses included Blood Uria Nitrogen (BUN), Creatinine (Cr), Hemoglobin (Hb), Hematocrit (Hct), Albumin (Alb), and Potassium (K) while the adequacy of hemodialysis measurement included weekly Kt/V and Normalized protein catabolic rate (nPCR).

#### Data collection procedures

Purposive sampling was used to obtain a sample of 54 OPLWH, who participated in the intervention phase of the study over a period of 6 months (between 1 April 2018 to 30 September 2018). Simple randomization was used to assign participants to either the experimental or the control group. The researcher sent information letters and copies of the study protocol to the administrators of the relevant medical units to ask them for permission to collect data. The medical units had a register of OPLWH which included patients’ telephone numbers; using this register, the researcher was able to invite potential participants to take part in the study by telephone. An advanced practitioner nurse in hemodialysis and two hemodialysis nurses who were trained as “supporters” helped to recruit participants, participated in monthly meetings and discussions with researcher and participants at the hemodialysis unit, and provided monitoring and consultation online. They also helped the researcher collect laboratory parameters from medical records at the hemodialysis unit. Participants were asked to provide information about demographic data and health status assessment by completing forms and undergoing routine blood testing at baseline, 3 and 6 months. Questionnaires and measurements were used to collect data from the participants at the hemodialysis unit by the researcher. The instruments included a demographic data form, the 9**-**item Thai Health Status Assessment Instrument, and blood chemistry analyses (BUN, Cr, Hb, Hct, K, Alb, Kt/V, and nPCR).

### Data analysis

Data were analyzed using SPSS programme version 17. Descriptive statistics were used to analyze participants’ demographic data and clinical characteristics. Chi-square test was used to test the difference in demographic data between the two groups at baseline. The independent t-test and one-way repeated measures ANOVA were applied to compare differences in mean scores for quality of life between the two groups. One-way repeated measures ANOVA was also used to test the interaction of the Home Telehealth Model within the two groups. ANOVA was used to detect the interaction of the model and statistically significant differences in laboratory results (BUN, Cr, Hb, Hct, K, Alb, Kt/V, nPCR) between the experimental and control groups at baseline and at 3 and 6 months.

## Results

### Information of participants

From Table 1, participants’ demographic data were analyzed at baseline, and there were no differences between the experimental and control groups in terms of gender, age, marital status, religion, education level, current occupation, people living together, regular contact person, and payment of medical expenses. Of all general characteristics, only income, duration of receiving hemodialysis, and illness with other disease showed a statistical difference between the intervention and control groups at baseline.

**Table 1:** Demographic characteristics of the participants in the experimental and control groups

Characteristic	Experimental group (*n* = 24)	Control group (*n* = 30)	χ^2^	*P*
	***n (%)***	*n**** (%)***		
**Gender**			0.035	0.851
Male	9 (37.5)	18 (60.0)		
Female	15 (62.5)	(40.0) 12		
**Age (years)**			0.381	0.827
69–60	(50.0) 12	(46.7) 14		
70–79	(29.2) 7	(36.7) 11		
> 80	(20.8) 5	(16.7) 5		
**Marital status**			0.613	434. 0
Single/Divorced/Widowed	(29.2) 7	(20.0) 6		
Married	(70.8) 17	(80.0) 24		
**Religion**			1.274	0.259
Buddhism	(95.8) 23	(100.0) 30		
Christianity	(4.2) 1	-		
**Education level**			2.796	0.247
Primary or lower	(62.5) 15	(43.3) 13		
Secondary education	(12.5) 3	(30.0) 9		
Higher Vocational	(25.0) 6	(26.7) 8		
**Current occupation**			0.561	0.454
Unemployed	(66.7) 16	(56.7) 17		
Employed	(33.3) 8	(43.3) 13		
**People living together**			2.035	0.154
Relative/Child	(37.5) 9	(20.0) 6		
Spouse	(62.5) 15	(80.0) 24		
**Regular contact person**			1.076	0.300
Relative/Child	(54.2) 13	(40.0) 12		
Spouse	(45.8) 11	(60.0) 18		
**Family income per month** (baht (THB)/month)			12.400	0.002*
< 3,000	(41.7) 10	(40.0) 12		
3,001–10,000	(20.8) 5	(23.3) 7		
> 10,000	(37.5) 9	(36.7) 11		
**Getting help with income**			2.078	0.149
Child	(58.3) 14	(76.7) 23		
Spouse/Relative/Other	(41.7) 10	(23.3) 7		
**Income sufficiency**			5.538	0.019*
Sufficient	(12.5) 3	(26.7) 8		
Insufficient	(87.5) 21	(73.3) 22		
**Payment of medical expenses**			0.635	0.425
Pay themselves	2 (8.3 (	(3.3) 1		
Health care coverage	(95.8) 22	(96.7) 29		

Data are n (%), *Abbreviations*: *χ*^*2*^ Chi-square test, Significant at * *P* < 0.05, 1 baht (THB) = 0.028 US Dollar (USD) or 1 US Dollar (USD) = 35.72 baht (THB)

In both groups, most participants were between 60–69 years old, and their education level was primary school. In addition, most of the participants were Buddhist. Most participants had insufficient income and received financial support from their children. However, both groups received payments from the health care coverage scheme for medical expenses.

Table 2 shows that most participants in both the experimental and control groups had received kidney replacement therapy or dialysis during the past 1–5 years. Moreover, both groups also had underlying diseases, for example hypertension, diabetes mellitus and heart disease, especially hypertension which was the highest percentage (70–80%) of the chronic diseases found in participants from each group. There were no differences in terms of demographic characteristics of the participants in receiving treatment when comparing the two groups.

**Table 2:** Demographic characteristics of the participants in the experimental and control groups classified by treatment being received

**Receiving treatment**	**Experimental group**	**Control group**	**χ**^**2**^	***P***
	***(n* = *24)***	***(n* = *30)***		
**Kidney replacement therapy**			3.375	0.066
Did not receive	(41.7) 10	(66.7) 20		
Received	(58.3) 14	(33.3) 10		
**Duration of receiving treatment**			26.375	0.000**
1 –5 years	(85.7) 12	(70.0) 7		
> 5 years	(14.3) 2	3 (30.0)		
Experienced kidney replacement therapy problems; had to return to hospital	(28.6) 4	(40.0) 4		
**Illness with other diseases**	19 (79.2)	27 (90.0)	26.421	0.000**
Diabetes mellitus	(50.0) 12	(50.0) 15		
Hypertension	(70.8)17	(80.0) 24		
Heart disease	(37.5) 9	(23.3) 7		
Lung disease	(4.2) 1	(3.3) 1		
Gouty arthritis	(8.3) 2	(20.0) 6		

Data are n (%), *Abbreviations*: *χ*^2^ Chi-square test, Significant at **P < 0.001

### Comparison of quality of life and laboratory parameters between and within the two groups

As shown in Table 3, before the intervention, the independent t-test results showed no statistically significant difference between the two groups in mean scores of laboratory parameters (*P* > 0.05). However, after 3 months, the independent t-test revealed a statistically significant difference between the two groups in the mean score of Kt/V (*P* < 0.05). In addition, 6 months after the intervention, the comparison of laboratory results between the two groups found a statistically significant difference in the mean hemoglobin (Hb) score (*P* < 0.05).

**Table 3:** Comparison of laboratory parameters between the two groups, *Mean* ± *SD*)

Parameters	Experimental group (*n* = 24)	Control group (*n* = 30)	*F*	*P*
Baseline				
BUN (mg/dL)	53.86 (21.61)	45.34 (23.52)	0.327	0.570
Cr (mg/dL)	9.67 (14.05)	6.22 (2.74)	3.204	0.080
Hb (g/dL)	10.40 (1.43)	12.34 (12.84)	1.949	0.169
Hct (%)	33.02 (4.55)	30.11 (7.18)	0.880	0.353
Alb (g/dL)	4.36 (1.88)	5.58 (9.06)	2.144	0.150
K (mEq/L)	4.42 (0.75)	5.53 (6.85)	1.901	0.174
Kt/V	1.75 (0.80)	1.81 (0.54)	0.151	0.700
nPCR (g/kg/day)	1.11 (0.27)	1.10 (0.31)	0.141	0.709
After 3 months				
BUN (mg/dL)	48.35 (21.10)	42.62 (14.66)	1.274	0.264
Cr (mg/dL)	6.53 (2.87)	6.69 (2.56)	0.196	0.660
Hb (g/dL)	10.95 (1.69)	14.52 (22.74)	2.203	0.144
Hct (%)	34.44 (4.78)	32.76 (6.17)	1.339	0.253
Alb (g/dL)	3.91 (0.60)	3.68 (0.63)	0.026	0.873
K (mEq/L)	4.35 (0.79)	4.18 (0.65)	0.051	0.822
Kt/V	1.59 (0.37)	1.67 (0.55)	4.250	0.044*
nPCR (g/kg/day)	0.99 (0.26)	0.99 (0.37)	0.921	0.342
After 6 months				
BUN (mg/dL)	50.26 (19.80)	46.07 (18.77)	0.563	0.457
Cr (mg/dL)	8.96 (9.70)	7.35 (2.94)	2.931	0.093
Hb (g/dL)	10.70 (1.25)	10.51 (1.96)	7.287	0.010*
Hct (%)	35.14 (8.44)	34.04 (9.13)	0.398	0.531
Alb (g/dL)	3.94 (0.48)	3.82 (0.67)	0.501	0.483
K (mEq/L)	4.40 (0.90)	5.52 (6.26)	1.872	0.177
Kt/V	1.66 (0.31)	1.66 (0.35)	0.005	0.942
nPCR (g/kg/day)	1.17 (0.74)	0.99 (0.23)	1.835	0.182

Kt/V = a measure of dialysis adequacy, K = clearance-the amount of urea your dialyzer can remove (liters/minute), t = time–the duration of treatment (minutes),V = volume-the amount of body fluid (liters)

*Abbreviations*: *BUN* Blood Uria Nitrogen (1 mg/dL = 0.3571 mmol/L), *Cr* Creatinine (1 mg/dL = 88.42 umol/L), *Hb* Hemoglobin (1 g/dL = 0.621 mmol/L), *Hct* Hematocrit (1% = 0.01 fraction), *Alb* Albumin (1 g/dL = 10 g/L), *K* Potassium (1 mEq/L = 1 mmol/L), *nPCR* Normalized protein catabolicrate

^*^P < 0.05 (Independent t-test)

Table 4 displays changes in the means of patient outcomes across three time periods. The repeated measured one-way ANOVA using the General linear model and Sphericity assumed for within-subjects and between-subjects effect were performed for the multiple comparison test. The comparison between the two groups at the three points of measurement showed no statistically significant difference in either quality of life or laboratory parameters. However, after 3 months, the quality-of-life scores for the mental health dimension showed statistically significant differences between the two groups (*P* < 0.05).

**Table 4:** Comparison of quality of life and laboratory parameters between and within the two groups (scores for the 9-item Thai Health Status Assessment Instrument, *Mean* ± *SD*)

**Variables**	Baseline		After 3 months		After 6 months		Between groups		Within groups		Interaction	
	Experimental Group (*n* = 24)	Control Group (*n* = 30)	Experimental Group (*n* = 24)	Control Group (*n* = 30)	Experimental Group (*n* = 24)	Control Group (*n* = 30)	*F*	*P*	*F*	*P*	*F*	*P*
**Quality of life: Physical health scores**	44.85 (8.09)	46.71 (7.03)	48.42 (8.92)	47.12 (12.69)	47.23 (11.33)	48.37 (8.98)	0.099	0.754	**0.865**	**0.424**	0.501	0.754
**Quality of life:** **Mental health scores**	45.86 (7.05)	46.21 (6.07)	49.89 (8.97)	48.68 (10.04)	48.40 (10.17)	49.60 (9.05)	0.004	0.954	**3.344**	0.039*	0.384	0.682
**BUN** (mg/dL)	(21.61) 53.86	45.34 (23.52)	(21.10) 48.35	(14.66) 42.62	(19.80) 50.26	(18.77) 46.07	2.723	0.105	**0.673**	**0.512**	0.132	0.876
**Cr** (mg/dL)	(14.04) 9.67	(2.74) 6.22	(2.87) 6.53	(2.55) 6.69	(9.70) 8.96	(2.94) 7.35	1.596	0.212	**0.661**	**0.518**	0.811	0.447
**Hb** (g/dL)	(1.42) 10.39	(12.83) 12.34	(1.69) 10.95	(22.74) 14.52	(1.25) 10.70	(1.96) 10.51	0.988	0.325	**0.424**	**0.656**	0.324	0.724
**Hct** (%)	(4.55) 33.02	(7.17) 30.11	34.44 (4.78)	32.76 (6.17)	(8.43) 35.14	(9.13) 34.04	2.420	0.126	**2.711**	**0.071**	0.241	0.786
**Alb** (g/dL)	(1.87) 4.36	(9.06) 5.58	(0.60) 3.91	(0.63) 3.68	(0.48) 3.94	(0.67) 3.82	0.254	0.617	**1.324**	**0.271**	0.489	0.615
**K** (mEq/L)	(0.75) 4.42	(6.85) 5.53	(0.79) 4.35	(0.65) 4.18	(0.90) 4.40	(6.26) 5.52	1.100	0.299	**0.513**	**0.601**	0.423	0.656
**Kt/V**	1.75 (0.79)	(0.54) 1.81	(0.37) 1.59	(0.55) 1.67	(0.31) 1.66	(0.35) 1.66	0.193	0.662	**2.066**	**0.132**	0.445	0.642
**nPCR** (g/kg/day)	(0.27) 1.11	1.10 (0.31)	(0.26) 0.99	(0.37) 0.99	(0.73) 1.17	(0.23) 0.99	0.310	0.580	**0.702**	**0.498**	1.653	0.197

Multiple comparison of variables of three time periods using Post Hoc* Test.* Significant at * *P* < 0.05

Kt/V = a measure of dialysis adequacy, K = clearance-the amount of urea your dialyzer can remove (liters/minute), t = time–the duration of treatment (minutes), V = volume-the amount of body fluid (liters)

*Abbreviations*: *BUN* Blood Uria Nitrogen (1 mg/dL = 0.3571 mmol/L), *Cr* Creatinine (1 mg/dL = 88.42 umol/L), *Hb* Hemoglobin (1 g/dL = 0.621 mmol/L), *Hct* Hematocrit (1% = 0.01 fraction), *Alb* Albumin (1 g/dL = 10 g/L), *K* Potassium (1 mEq/L = 1 mmol/L), *nPCR* Normalized protein catabolicrate

## Discussion

This study evaluated whether a hemodialysis nursing team integrated with a home telehealth model was a feasible method of holistic care delivery to maintain quality of life and improve health outcomes among OPLWH. The results confirm that the integrated intervention is feasible for improving the participants health outcomes. Telehealth and inter-professional care can be successfully implemented with meaningful engagement as part of the care system while telehealth delivered and supported by an inter-professional team has been shown to be a feasible care delivery strategy for CKD patients [14]. Advances in health technology over recent decades have kindled interest in the possibility of using home telehealth programs to extend human resources, improve access to services, and minimize the costs of care [15].

Before the COVID-19 pandemic, there were limitations related to remote care that created barriers to patient access to continuum care via telehealth, and these limitations disincentivized renal nurses to offer telehealth as an option. However, providing and receiving tele-visiting became the norm for nurses, healthcare teams, and patients when social distancing, stay-at-home, and other hospital policies were introduced in 2020 due to the COVID-19 pandemic. The utilization of the home telehealth model should be promoted and expanded for its ability to promote equal access to telehealth and the opportunity for CKD patients living with hemodialysis to receive continuing care outside hospitals. Both inpatient and outpatient practices had to quickly adapt to telehealth [30], and the development of home telehealth services has since become an essential and valuable aspect of care, allowing patients with advanced conditions to remain at home and in their own communities, although it is still the case that when they experience problems, they may feel unsure about who they can contact, or how. Nevertheless, the use of telehealth services can help to empower individuals in terms of their experiences with life-limiting illness, and the experiences of their caretakers, by facilitating the provision of real-time communication between patients and healthcare providers [31]. It can also be used to complement transitions from acute services based on patients’ needs [32].

In this study, the participants who received the home telehealth model showed no significant effects observed in terms of their quality of life or clinical outcomes over 6 months (*P* > 0.05). Comparison within the intervention group between two time measurements (at baseline vs after 3 months) found statistical evidence of improvement in the mean scores of health outcomes (BUN: 53.86 ± 21.61 vs 45.34 ± 21.10; Cr: 9.67 ± 14.04 vs 6.53 ± 2.87; Hb: 10.39 ± 1.42 vs 10.95 ± 1.69; Hct: 33.02 ± 4.55 vs 34.44 ± 4.78; Alb: 4.36 ± 1.87 vs 3.91 ± 0.60; and K: 4.42 ± 0.75 vs 4.35 ± 0.79) and patients’ quality of life (physical health scores: 44.85 ± 8.09 vs 48.42 ± 8.92 and mental health scores: 45.86 ± 7.05 vs 49.89 ± 8.97). After 6 months, the comparison within the intervention group showed increases in the mean scores of Hct (34.44 ± 4.78vs 35.14 ± 8.43), Kt/V (1.59 ± 0.37 vs 1.66 ± 0.31), and nPCR (0.99 ± 0.26 vs 1.17 ± 0.73). According to the three-time measurements of quality of life and laboratory parameters, there was no statistical difference between the two groups (*P* > 0.05). However, regarding the comparison using the independent t-test, after 3 months and 6 months, some of the laboratory (Kt/V and Hb) results showed statistically significant differences between the two groups (*P* < 0.05) while some of the tests were not significant (BUN, Cr, Hct, K, Alb, and nPCR) (*P* > 0.05). In addition, after 3 months repeated measurement, quality of life, in terms of the mental health scores, showed statistically significant differences between the two groups (*P* < 0.05).

Further investigation might be needed in terms of the relationships between each laboratory result and the intervention. This finding is contradictory to the findings of previous studies. According to Minatodani, Chao, and Berman, home telehealth for high-risk dialysis patients has demonstrated improvements in the cost-effectiveness of health outcomes over 21 months [33]. In addition, a randomized controlled study on the use of home telehealth in promoting illness self-management demonstrated an improvement in health outcomes and cost-effectiveness for high-risk dialysis patients [34]. Home telehealth has also demonstrated improved health outcomes and cost-effectiveness for high-risk patients with ESRD, using remote technology for home health monitoring with support from remote care nurses [34]. Another study reported a statistically significant reduction in depression, anxiety, and stress due to telehealth interventions [35]. In contrast, the biomarkers predicted patients’ clinical outcomes. Standardized Kt/V was a significant indicator for the adequacy of the dialysis and was related to a reduction in clinical complications of dialysis patients, such as hypocalcemia, hyperphosphatemia, and anemia [36]. It was also suggested that poorer laboratory indicators demonstrated an inability to control mineral bone density and anemia [12]. The participants received hemodialysis treatment 2–3 times per week, and they needed to evaluate their hemodialysis adequacy following the KDOQI Clinical Practice Guidelines for Hemodialysis Adequacy: 2015 update [36]. Dialysis dose has been reported to have great significance for the outcome of hemodialysis treatment. Many studies have shown a relationship between dialysis dose, measured as Kt/V, and morbidity and mortality among hemodialysis patients [36]. In the case of inadequate Kt/V, patients needed to increase the dose of dialysis. In addition, the lower nPCR indicated that patients needed to increase their protein intake, or they would be at a risk for malnutrition. Patients who had low serum levels of albumin and creatinine clearance may report low quality of life and inadequacy of dialysis [17, 36].

These results suggest that at 3 months after the intervention, the experimental group achieved a higher quality of life than the control group along both physical and mental dimensions. In the experimental group, the mean quality of life from the mental health scores showed a more positive increase than for the control group at three points of measurement. However, the success of home telehealth depends strongly on patient adherence to the prescribed program [6, 17]. Home telehealth with remote care nurse (RCN) support can promote health behavioral change, resulting in better outcomes. Moreover, home telehealth self-monitoring with RCN support can effectively empower patients to take a more active role in their healthcare, indirectly improving quality of life for people living with chronic illness [18]. Unfortunately, we did not detect a statistically significant difference in quality of life between the two groups using repeated independent t-test and one-way repeated ANOVA. This might be because the six-month duration was not sufficient, or because the sample group was simply too small to observe a significant difference. In addition, 50% of participants in both groups were between 60–69 years old, had a low education level, needed financial support, and depended on their family members. These demographic characteristics could be indirect factors in reducing patients’ ability for maintaining adherence behavior in management with hemodialysis complications at home. Moreover, longtime management with hemodialysis and having comorbidities such as hypertension, diabetes mellitus, and heart disease can have negative impacts by increasing morbidity and mortality rates, reducing quality of life, and resulting in inadequate symptom management at home [24]. According to Table 2, the difference in duration of receiving hemodialysis treatment, especially for patients with CKD stage 3–5, could result in poorer clinical outcomes and higher risk of emergency department visit, hospitalization, and death. This is related to the negative impacts of prolonged hemodialysis cycles for people living with ESRD, and fluctuations in terms of their cognitive and physical wellbeing, as well as in their emotional state [14]. Increased age, history of diabetes, longer dialysis vintage, and lower Kt/V were independently associated with frailty [37]. Frailty is also extremely common and influences serious clinical outcomes among older hemodialysis patients [37]. Integrating a home telehealth model with nurse oversight into holistic care for OPLWH can help them maintain their quality of life and improve their health outcomes. Moreover, the literature related to telehealth and kidney disease care has strongly reflected high patient satisfaction [30]. The results of this study show that integrating home telehealth with nurse oversight into holistic care for OPLWH has acceptable feasibility in providing a higher quality of life, as well as in terms of relative improvements in some biometric outcomes and the quality of dialysis. Therefore, it will be highly beneficial to establish such a model in renal units. However, the effectiveness of different telehealth models may need to be evaluated before implementation.

### Strengths and limitations

Like any research, our study has both limitations and strengths. This is the first study of its kind in Thailand. Its main strength is the study’s implementation site, namely a hemodialysis center in an urban setting where the prevalence of OPLWH is relatively high.

On the other hand, our study had two limitations. Our relatively small sample size is likely to limit the external validity of the study. We should also recognize that the participants were OPLWH who might be unable to access particular technologies, or who may be unfamiliar with using technologies such as web applications. To solve this problem, we asked caregivers to stay with participants during the training and to be present during consultations in the 6-month intervention period (monthly routine online visits and telephone consulting), in order to help participants to access the web application and to support them when they needed help. Other studies have also found that more comprehensive interventions can require a caregiver or other person to assist patients [30].

However, those who live alone and have inadequate social support might have poorer self-management behaviors [17, 38] and might not gain as much benefit from a telehealth model in terms of disparities in accessing telehealth for patients having lower socio-economic status. Moveover, patients with limited health literacy or geriatric symptoms, such as hearing impairment, visual problems, and fingers or bodily shaking, may not be able to operate the video calls or may not have the technical capability. In addition, it is possible that these factors affect the ability of some to have a serious discussion with clinicians during the time that they communicate via video telehealth or phone. In addition, older persons who live alone may not be able to maintain their engagement in management with self-care, medical regimens, and self-monitoring, affecting their ability to effectively report about their clinical progression [21].

Furthermore, the study was conducted at a time when third generation (3G) internet coverage was offered in Thailand. This was suitable for participants who owned modern smartphones and lived in urban areas with constant high-speed internet connection but may have presented limitations for participants in more rural areas or those who did not have access to the most up-to-date technology. To mitigate these problems, the researcher provided all participants with a mini-iPad and free Wi-Fi for 6 months so that they could regularly access the home telehealth program. Currently, fifth generation (5G) internet coverage is standard in Thailand, and Thai health policy is focusing on the digital transformation concept to ensure the vast majority of users, including the older population, are familiar with accessing internet communication in both urban and rural areas.

### Implications for clinical practice

Due to limitations on renal nurses visiting patients at home and the transforming of the digital health system after the COVID-19 pandemic, older persons undergoing hemodialysis may suffer with clinical complications at home and require clinical nurse specialists to provide them with continuing renal care. Integrating a home telehealth model for this population is a feasible response, enhancing their quality of life and improving their health outcomes. The value of a home telehealth self-monitoring model with nurse oversight has been demonstrated with positive results in terms of reducing clinical complications. Despite less contact with homecare facilities due to shortages of clinical nurse specialists visiting patients at home, OPLWH experienced sustained quality of life and improved clinical outcomes after receiving the integrated home telehealth model.

In the future**,** renal nurses in dialysis units should emphasize the use of a telehealth model to promote the physical and mental health of their patients. Effective digital health innovations for virtual care, such as the home telehealth model implemented in this study, can provide effective solutions to the problem of a shortage of renal nurse specialists, and possibly decrease care costs for patients. Despite the study limitations, however, we recommend the use of a home telehealth model for OPLWH, based on its potential for increasing quality of life and reducing the risk of clinical complications by improving patients’ health outcomes. Further studies within different settings, populations, and cultural contexts are recommended in order to confirm the effectiveness of the home telehealth model. In addition, improving the cost effectiveness of future studies should focus on larger sample sizes, 1-year interventions, and intensive retraining sessions for patients, caregivers, and renal clinicians. An expansion of the criteria of reimbursement and regulation for patients who receive telehealth services is needed. Telephone calls may be appropriate for patients of lower socio-economic status, or those having poor internet access and low levels of technological literacy.

## Conclusions

Our findings confirm that the implementation of a home telehealth model can yield positive changes in all aspects, including quality of life and health outcomes, for OPLWH. However, our findings are based on a short-term intervention over a six-month period. The long-term benefits of such a program should also be evaluated, and its economic impact on the delivery of healthcare services should be assessed. Our integrated holistic home telehealth model was an effective strategy to increase health-related quality of life and health outcomes over a 6-month implementation period. Our study provides evidence in favour of the feasibility of a home telehealth model for OPLWH after they have received training alongside their standard care. Such a model may potentially reduce workloads for renal nurses and improve routine care, enhancing health-related quality of life and reducing complications among OPLWH.

## Data Availability

The datasets used and analyzed during the current study are available from the corresponding author on reasonable request. The statistics were checked prior to submission by an expert statistician and state their name and email address.

**Table:** 

**Status of data and materials**	**Statement**
Data available on request due to privacy/ethical restrictions	The data that support the findings of this study are available on request from the corresponding author, [Pungchompoo Wanicha: wanicha.p@cmu.ac.th]. The data are not publicly available due to [restrictions e.g. their containing information that could compromise the privacy of research participants].
Data generated at a central, large-scale facility, available upon request	Raw data were generated at [facility name]. Derived data supporting the findings of this study are available from the corresponding author [Pungchompoo Wanicha: wanicha.p@cmu.ac.th] on request.
Data available within the article or its supplementary materials	The authors confirm that the data supporting the findings of this study are available within the article.
Data available on request from the authors	The data that support the findings of this study are available from the corresponding author, [Pungchompoo Wanicha: wanicha.p@cmu.ac.th], upon reasonable request.

## Abbreviations

CKD: Chronic kidney disease
RRT: Renal replacement therapy
PD: Peritoneal dialysis
HD: Hemodialysis
KT: Kidney transplantation
ESRD: End-stage renal disease
OPLWH: Older persons living with hemodialysis
eGFR: Estimated glomerular filtration rate
BUN: Blood Uria Nitrogen
Cr: Creatinine
Hb: Hemoglobin
Hct: Hematocrit
Alb: Albumin
K: Potassium
nPCR: Normalized protein catabolicrate
Kt/V: A measurement of dialysis adequacy
URR: Urea reduction ratio
